# Higher Diplomas

**Published:** 1919-09-06

**Authors:** 


### HIGHER DIPLOMAS.

**Royal College of Physicians of London** grants its membership after examination to graduates in medicine of recognised Universities, or to licentiates of the College, being above the age of twenty-five years, who do not engage in trade, do not dispense medicine, and who do not practise in partnership. The examination, which is held in January, April, July, and October, is partly written and partly oral. The fee for the membership is £42, but if the candidate is a licentiate the fee is the difference between what he has already paid for the licence and £42. In either case £6 6s. is paid before examination. The fellowship is elective.

**Royal College of Physicians of Edinburgh** admits to its membership any licentiate of a British or Irish College of Physicians, or graduate in medicine of a University within the British Empire, approved by the Council, over twenty-four years of age, after examination in medicine and therapeutics and one or more of the following subjects selected by the candidate: (1) One or more departments of medicine specially professed, (2) psychological medicine, (3) general pathology and morbid anatomy, (4) medical jurisprudence, (5) public health, (6) midwifery, (7) diseases of women, (8) diseases of children, (9) tropical medicine. The fee to be paid by a licentiate is twenty guineas, by others thirty-five guineas. A member of three years' standing may be elected a Fellow. The fee is £64 18s.

**Royal College of Surgeons of Edinburgh**

The Fellowship is granted, after examination, to any registered practitioner who is twenty-five years of age, and has been in practice for two years, on petition being granted. The examinations are written, oral, and practical. The fee is £35 to those who hold the diploma of licentiate of the College, and £45 to others (no stamp duty is payable on the diploma). Apply for particulars to Mr. D. L. Eadie, 49 Lauriston Place, Edinburgh.

**Royal Faculty of Physicians and Surgeons of Glasgow.**—Registered practitioners are admitted to the Fellowship by examination and by subsequent election. Four examinations are held annually (January, April, July, October). Fourteen days' notice must be given. The fee is £30 unless the candidate desires to qualify to hold office, when it is £50. In the case of a licentiate of the faculty, £25 and £15 respectively.

**Royal College of Surgeons in Ireland Fellowship.**—(1) The examination for the Fellowship is divided into two parts—namely, the primary and the final. (2) The subjects of the Primary examination are anatomy (including dissections), physiology, and histology. The examination is partly written, partly *viva voce*, and partly practical. Candidates must pass in all the subjects at one examination. (3) The subjects of the Final examination are surgery (including surgical anatomy) and pathology. The examination is partly written and partly *viva voce*, and includes the examination of patients and the performance of operations on the dead body. Candidates must pass in all the subjects at one examination. (4) The examinations are held three times in each year. (5) Special examinations will not be granted by the Council under any circumstances. (6) Candidates are required to lodge their applications, certificates, and receipts for fees with the Registrar at least seven days before the date of the examination.

